# Aberrant Location of Inhibitory Synaptic Marker Proteins in the Hippocampus of Dystrophin-Deficient Mice: Implications for Cognitive Impairment in Duchenne Muscular Dystrophy

**DOI:** 10.1371/journal.pone.0108364

**Published:** 2014-09-26

**Authors:** Elżbieta Krasowska, Krzysztof Zabłocki, Dariusz C. Górecki, Jerome D. Swinny

**Affiliations:** 1 Laboratory of Cellular Metabolism, Nencki Institute of Experimental Biology, Warsaw, Poland; 2 School of Pharmacy and Biomedical Sciences, Institute for Biomedical and Biomolecular Sciences, University of Portsmouth, Portsmouth, United Kingdom; Institut National de la Santé et de la Recherche Médicale (INSERM U901), France

## Abstract

Duchenne muscular dystrophy (DMD) is a neuromuscular disease that arises from mutations in the dystrophin-encoding gene. Apart from muscle pathology, cognitive impairment, primarily of developmental origin, is also a significant component of the disorder. Convergent lines of evidence point to an important role for dystrophin in regulating the molecular machinery of central synapses. The clustering of neurotransmitter receptors at inhibitory synapses, thus impacting on synaptic transmission, is of particular significance. However, less is known about the role of dystrophin in influencing the precise expression patterns of proteins located within the pre- and postsynaptic elements of inhibitory synapses. To this end, we exploited molecular markers of inhibitory synapses, interneurons and dystrophin-deficient mouse models to explore the role of dystrophin in determining the stereotypical patterning of inhibitory connectivity within the cellular networks of the hippocampus CA1 region. In tissue from wild-type (WT) mice, immunoreactivity of neuroligin2 (NL2), an adhesion molecule expressed exclusively in postsynaptic elements of inhibitory synapses, and the vesicular GABA transporter (VGAT), a marker of GABAergic presynaptic elements, were predictably enriched in *strata pyramidale* and *lacunosum moleculare*. In acute contrast, NL2 and VGAT immunoreactivity was relatively evenly distributed across all CA1 layers in dystrophin-deficient mice. Similar changes were evident with the cannabinoid receptor 1, vesicular glutamate transporter 3, parvalbumin, somatostatin and the GABA_A_ receptor alpha1 subunit. The data show that in the absence of dystrophin, there is a rearrangement of the molecular machinery, which underlies the precise spatio-temporal pattern of GABAergic synaptic transmission within the CA1 sub-field of the hippocampus.

## Introduction

Duchenne muscular dystrophy (DMD) [1] is a neuromuscular disease that arises due to the absence of the protein dystrophin [2]. Dystrophin functions to provide a scaffold for the formation of a complex of dystrophin-associated proteins (DAP), including dystroglycan and syntrophin, which provide a link between the cytoskeleton, cell membrane and extracellular matrix components [3]. Disruption of this protein complex results in not only muscle degeneration but also the added burden of non-progressive cognitive impairment, which DMD patients suffer from, [1] [4], [5]. This impaired cognitive performance in DMD patients, primarily of developmental origin, highlights the central role that dystrophin contributes firstly to brain development as well as coordinated neuronal activity in adulthood. However, the precise pathological mechanisms underlying DMD-dependent cognitive impairment are poorly understood.

This apparent multi-factorial role of dystrophin arises from its complex expression patterns due to alternative promoter usage and extensive alternative splicing, especially in brain [6]–[9]. As a result, the brain contains the highest number of dystrophin isoforms. Importantly, many of these dystrophin isoforms are expressed in brain areas associated with cognitive functions. Indeed, the expression patterns of dystrophins/DAP complexes brain region and cell-type specific; CA pyramidal neurones contain 427 kDa dystrophin and α1-syntrophin whereas dentate granule cells have 71 kDa dystrophin and α2-syntrophin [7], [8], [10], [11]. These different dystrophin/DAP complexes assemble at post-synaptic densities [12]. Alterations of the expression of specific isoforms in hippocampal neurons in response to kainate (Gorecki *et al.,* 1998) and in sympathetic neurons following axotomy (Zaccaria *et al.,* 1998), as well as changes in synaptic plasticity in dystrophic mdx mice (Vaillend *et al.,* 1999) suggest dystrophin involvement in assembly–disassembly and/or preservation of the postsynaptic apparatus. Moreover, β-dystroglycan is a matrix metalloproteinase-9 substrate digested in response to enhanced synaptic activity [13]. Increasing evidence points to dystrophin/DAP complexes having a central role in regulating the molecular machinery of synapses and thus having a central role in synaptic transmission. A large body of work mainly using the mdx mouse model of DMD indicates that the lack of dystrophin results in a decrease in the synaptic quotient of GABA-A receptors (GABA_A_Rs) [14], and altered GABAergic synaptic transmission [15]–[18].

While the importance of dystrophin in contributing to the anchoring of neurotransmitter receptors at post-synaptic densities is unequivocal, its role in synapse formation has attracted less attention. This is surprising given the fact that β-dystroglycan is a physiological ligand for neurexins [19]. Neurexins and neuroligins are synaptic cell-adhesion molecules that connect presynaptic and postsynaptic neurons at synapses and thus occupy a central role in synapse formation [20]. As such, any dystrophin-deficiency evoked alterations in neurxin-neuroligin functions are likely to impact on synapse formation. Such malformation of synapses will have a dramatic effect in brain regions, which rely on the precise spatial patterning of synaptic inputs for the coordinated network activity underlying that brain region’s contribution to the cognitive function. The importance of synapse location is exemplified in the CA1 region of the hippocampus. Excitatory synapses on individual CA1 pyramidal neurons are organised onto precise sub-cellular dendritic domains located in either *strata oriens, radiatum* or *lacunosum moleculare*, depending on the origins of their axons. In contrast, GABAergic synaptic inputs onto individual pyramidal neurons, arising from up to 21 functionally distinct inhibitory interneurons [21], are preferentially targeted to perisomatic pyramidal cell surfaces located in *stratum pyramidale* and to a lesser extent, to dendritic surfaces in *stratum lacunosum moleculare*. This precise stratification of functionally diverse synaptic inputs onto distinct sub-cellular domains of individual pyramidal cells is essential for the integration of the heterogeneous information conveyed during synaptic transmission and thus a prerequisite for the coordinated network activity occurring within the hippocampus that underlies cognition. Recent evidence indicates that inhibitory synapses on CA1 pyramidal neurons of mdx mice undergo reorganisation [22]. In the current study, we build on these finding by demonstrating that there is a cell-type specific re-distribution of inhibitory inputs onto CA1 pyramidal neurons in this mouse model of DMD.

## Materials and Methods

All procedures involving experimental animals were performed in accordance with the Animals (Scientific Procedures) Act, 1986 (UK) and associated procedures under Project License number PPL 70/7094. Every effort was made to minimise any pain or discomfort to the animals.

### Animals

Three month old C57Bl/10, wild-type (WT), as well as Dmd^mdx^ (mdx) [23] and Dmd ^Gt(ROSAβgeo)1Mpd^ (mdx^βgeo^) [24] dystrophin-mutant mice were used. The mdx lacks the full-length dystrophins due to a point mutation in exon 23 while mdx^βgeo^ is dystrophin-null. Animals were maintained in a 12 h light/dark cycle and fed normal diet and water *ad libitum*. A total of 8 mice from each genotype were used for qualitative and quantitative analyses.

### Tissue preparation

Animals were perfusion-fixed as follows: anaesthesia was induced with isoflurane and maintained with pentobarbitone (1.25 mg/kg of bodyweight; i.p.). The animals were perfused transcardially with 0.9% saline solution for 1 minute, followed by 12 minutes with a fixative consisting of 1% paraformaldehyde, 15% v/v saturated picric acid, in 0.1 M phosphate buffer (PB), pH 7.4. The brains were kept in the same fixative solution overnight at 4°C. Coronal sections of the dorsal hippocampus, 60 µm thick, were prepared on a Vibratome and stored in 0.1 M PB containing 0.05% sodium azide.

### Immunohistochemical reactions

Immunohistochemical procedures were according to those used in [25]. Five animals from each genotype were used to confirm the native immunoreactivity patterns whilst three animals from each genotype were used for quantitative analyses. A proteolytic antigen retrieval method was used to localise membrane–bound epitopes [26], [27] for neurligin2 (NL2) and GABA_A_R alpha1 immunoreactivity. Briefly, the tissue sections were incubated at 37°C for 10 minutes in 0.1 M PB followed by 15 minutes in 0.2 M HCl containing 1 mg/ml pepsin (Sigma, UK) after which they were washed thoroughly in Tris-buffered saline containing 0.3% triton (TBS-Tx) for 30 minutes. Non-specific binding of secondary antibodies was blocked by incubating sections with 20% normal horse serum for 2 hours at room temperature. The tissue sections were incubated with cocktails of primary antibodies diluted in TBS-Tx, overnight at 4°C. See Table 1 for antibody details and their specificity references. After washing with TBS-Tx, sections were incubated in a mixture of appropriate secondary antibodies conjugated with either Alexa Fluor 405 (Jackson ImmunoReserach) Alexa Fluor 488 (Invitrogen, Eugene, OR), indocarbocyanine (Cy3; Jackson ImmunoResearch), and indodicarbocyanine (Cy5; Jackson ImmunoResearch), all diluted 1∶1000, for 2 hours at room temperature. Sections were washed in TBS-Tx and mounted in Vectashield mounting medium (Vector Laboratories, Burlingame, CA).

**Table 1:** Details of antibodies used in the study.

Primary antibodies	Species (raised in)	Source/code	Dilution	Specificity/reference
Cannabinoid receptor 1	guinea pig	Frontier Science (Hokkaido,Japan), # CB1-GP-Af530	1∶3000	[64]
GABA_A_R alpha1 subunit	rabbit	Werner Sieghart, Antigensequence -α_1N_ amino acids1–9, Rabbit # 21/7, bleed# 04/10/1999.	1∶10000	[25]
Kv2.1	mouse	Neuromab	1∶1000	[65]
Neuroligin2	guinea pig	Frontier Science(Hokkaido, Japan),# Nlgn2-GP-Af760	1∶250	[66]
Parvalbumin	goat	Swant (Marly 1,Switzerland) # PVG 214	1∶2000	[67]
Somatostatin	mouse	GeneTex, # GTX71935	1∶500	Labelling pattern as publishedwith other antibodies
VGAT	Guinea-pig	Synaptic Systems# 131004	1∶1000	[68]
VGLUT3	goat	Frontier Science (Hokkaido, Japan),# VGLuT3-Go-Af870	1∶500	[39]

### Image acquisition

Sections were examined with a confocal laser-scanning microscope (LSM710; Zeiss, Oberkochen, Germany) using either a Plan Apochromatic 20x DIC oil objective (NA0.8). a Plan Apochromatic 63x DIC oil objective (NA1.4) or a Plan Apochromatic 100x DIC oil objective (NA1.46). Z-stacks were used for routine evaluation of the labelling. All images shown represent a single optical section. These images were obtained using sequential acquisition of the different channels to avoid cross-talk between fluorophores, with the pinholes adjusted to one airy unit for all channels. Images were processed with the software Zen2008 Light Edition (Zeiss, Oberkochen, Germany) and exported into Adobe Photoshop. Only brightness and contrast were adjusted for the whole frame, and no part of a frame was enhanced or modified in any way.

### Quantification of either the density of individual immunoreactive clusters or the intensity of fluorescence within the CA1 region of WT and dystrophin-deficient mice

Our qualitative investigations revealed that most of the changes in the immunoreactivity patterns manifested in *strata pyramidale* and *radiatum*. Since these two layers of the hippocampus represent the sites of predominantly inhibitory and excitatory synaptic inputs, respectively, we restricted our quantifications to these layers in line with the focus of the study on the role of dystrophin in influencing the location of synaptic inhibitory marker proteins within defined hippocampal *strata*. Immunoreactivity for the voltage-gated potassium channel Kv2.1 was used to delineate the somatic and proximal apical dendritic domains of CA1 pyramidal neurons [28], [29] and which are the conventional locations for inhibitory synapses [30] and thus immunoreactivity for proteins which are expressed within such populations of synapses. The levels of immunoreactivity within such somato-dendritic locations were compared to that within the distal region of *stratum radiatum*, which contains almost exclusively excitatory input [30] and predictably contains significantly lower levels of immunoreactivity for inhibitory synaptic marker proteins.

The quantitative method used is according to [25]. For immunoreactivity patterns which presented as individual clusters (neuroligin2; vesicular glutamate transporter 3; GABA_A_R alpha1 subunit), the cluster density (number of clusters per µm^2^) was determined. Immunoreactivity patterns for which individual profiles were less distinct (VGAT; CB1, PV), densitometry of the signal in the form of fluorescence intensity was determined. In all cases, the perfusion fixation, reacting and imaging of tissue from WT and dystrophin-deficient mice was performed under identical conditions. For all quantitative analyses, a minimum of 3–5 tissue sections per animals, 3 animals in total were used. The imaging and quantification was performed as follows: within a tissue section, 3 fields of view (FOV) were randomly selected within the CA1 layer of interest. Z-stacks consisting of three optical sections spaced 2 µm apart in the Z plane were acquired for each FOV. The dimensions of each optical section were 85 µm×85 µm×1 µm in the X-Y-Z planes. Since the intention was to compare the levels of immunoreactivity across the different genotypes, raw, unprocessed images from the different genotypes, obtained under identical imaging conditions, were used for quantification purposes. Optimal imaging conditions were always initially obtained using specimens from WT animals and then used unchanged for those from mdx and mdx^βgeo^ animals. Within an optical section, the number of immunoreactive clusters was manually counted or the total fluorescence intensity determined as a measure of mean pixel intensity using ImageJ software. A value for each FOV was obtained by computing the average from the optical sections contained within a FOV. The means ± SD (cluster density or fluorescence intensity, arbitrary units, AU) for all FOV between sections and between genotypes was compared for statistical differences using Kruskal–Wallis one-way analysis of variance. These values were then pooled since there were no statistical differences (P>0.05) between the values for FOV between sections and between animals for a particular genotype. The average value from all FOVs and sections per animal were computed for individual animals. This average value for an individual animal was then considered an N of 1.

### Quantification of the number of parvalbumin and somatostatin-immunopositive cells within the CA1 sub-field of WT and dystrophin-deficient mice

The method used was according to [31]. A minimum of 3 tissue sections per animal, 3 animals in total were used. Using a 20x objective and 0.6 digital zoom, fields of view (FOV) were acquired across the entire CA1 region for a particular 60 µm-thick tissue section. For each FOV, a Z-stack was obtained throughout the full extent of the tissue section. The Z-stack consisted of optical sections measuring 425 µm×425 µm in the X & Y planes and 5 µm in the Z plane. In all cases, signal for parvalbumin and somatostatin was sequentially acquired using sequential acquisition of the different channels with acquisition parameters identical for all genotypes. The number of cells per tissue section and then the average number per animal were determined. The mean ± SEM was then calculated for N = 3 WT, mdx and mdx^βgeo^ animals.

### Statistical analysis

All quantitative data are presented as the mean ± SEM, unless otherwise stated. The data were tested for normality using a Shapiro-Wilk test. One-way analysis of variance (ANOVA) was used to test for significant differences between the mean values obtained for the 3 genotypes with a Tukey's post-hoc test used to assess differences between specific groups. In all cases, GraphPad Prism6 was used for statistical analyses and the graphical presentation of the data.

## Results

In this study, we exploited molecular markers of pre- and postsynaptic elements as well specific interneuron sub-classes to investigate the potential role of dystrophin in influencing the location of putative inhibitory synapses within specific layers of the CA1 region of the hippocampus.

### Altered distribution of the postsynaptic marker protein neuroligin2 (NL2) in the CA1 region of the hippocampus

**Figure 1:**
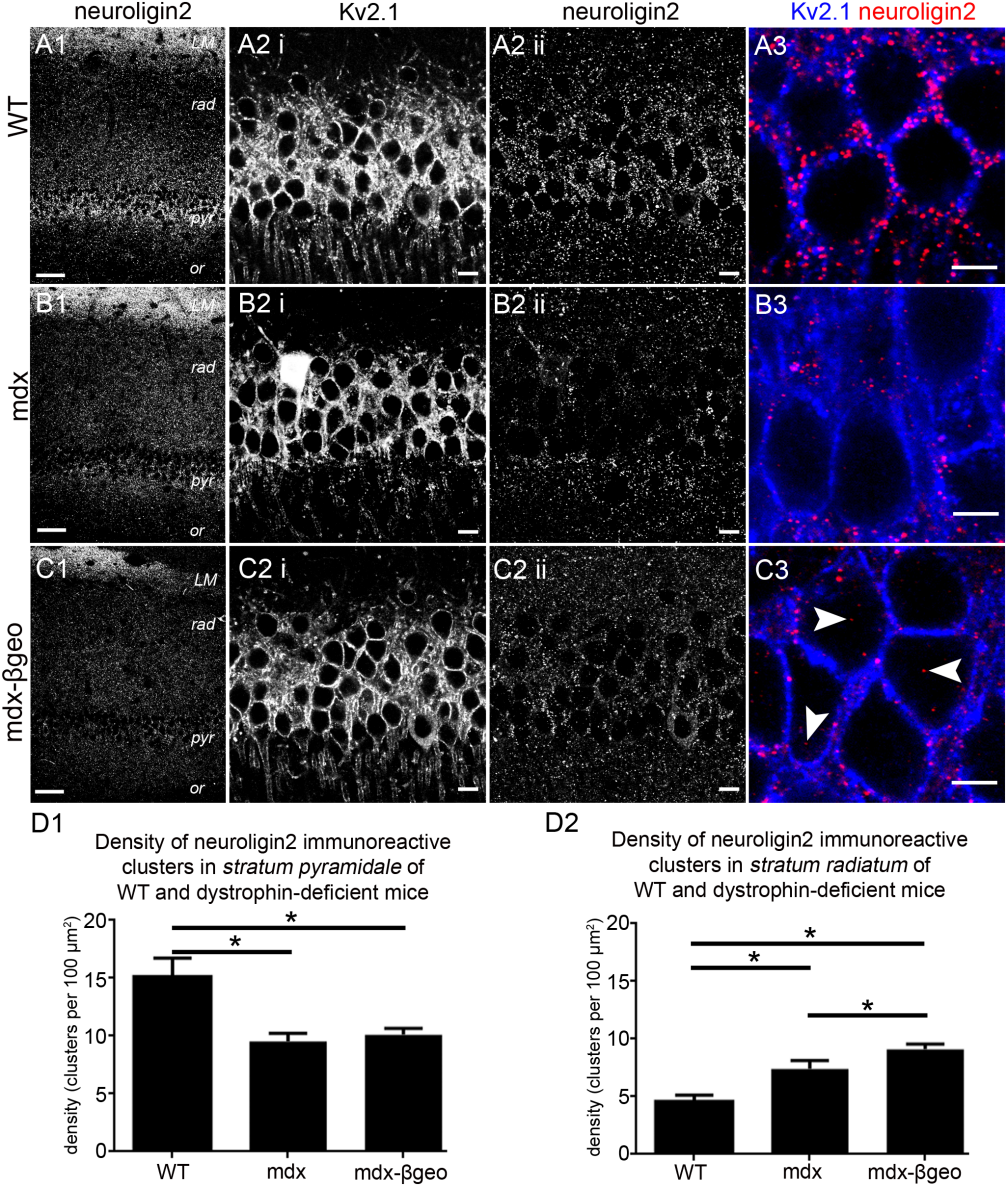
Localisation and quantification of NL2 immunoreactivity within the hippocampus of WT and dystrophin-deficient mice, reacted and imaged under identical conditions. (A 1) shows the stereotypical pattern of NL2 immunoreactivity within the CA1 region of tissue from WT animals. NL2 immunoreactivity is concentrated in *strata pyramidale* and *lacunosum moleculare* with considerably lower levels of signal in *stratum radiatum*, particularly within the distal parts of this layer. In contrast, significantly lower levels of NL2 immunoreactivity were evident within the *stratum pyramidale* of (B 1) mdx and (C 1) mdx^βgeo^ mice. (A 2 i) shows immunoreactivity for KV2.1 within *stratum pyramidale* of WT tissue. Kv2.1 is used to delineate the somato-dendritic plasma membranes and thus the major sites of inhibitory input onto pyramidal neurons. (A 2 ii, 3) shows that that within this layer, NL2 immunoreactivity presented as individual clusters located exclusively on somato-dendritic cell surfaces. (B 2 i) and (C 2 i) show that the level and pattern of Kv2.1 immunoreactivity within tissue from mdx and mdx^βgeo^ mice respectively was indistinct to that of WT mouse. This indicates that comparable fields of view and focal planes were selected for tissue from all genotypes. (B 2 ii, 3) and (C 2 ii, 3) demonstrate the significant decrease in the density of NL2 immunoreactive clusters within the *stratum pyramidale* of mdx and mdx^βgeo^ tissue respectively. Numerous cytoplasmically located NL2 immunopositive clusters were also evident in tissue from mdx^βgeo^ mice (arrowheads) in comparison to WT in which virtually all clusters were located on Kv2.1 immunopositive plasma membranes. (D 1) quantification of the density of NL2 immunoreactive clusters in *stratum pyramidale* of WT, mdx and mdx^βgeo^ tissue. (D 2) quantification of the density of NL2 immunoreactive clusters in the *stratum radiatum* of WT, mdx and mdx^βgeo^ tissue. Bars represent the means and lines the SEM. *P<0.05; ANOVA with Tukey’s posthoc test; N = 3 animals. Scale bars (A1, B1, C1) 50 µm; (A2, B2, C2) 10 µm; (A3, B3, C3) 5 µm.

As a start, we used NL2 immunoreactivity to investigate the distribution of putative inhibitory synapses in the CA1 region of WT and dystrophin-deficient mice. NL2 is exclusively localised to inhibitory synapses [32] and functions in the assembly of the postsynaptic apparatus in inhibitory synapses [33], [34]. The majority of inhibitory synapses on CA1 pyramidal neurons are located perisomatically [30]. In accordance, NL2 within the CA1 region of the hippocampus from WT mice has been shown to be enriched in *stratum pyramidale* [31] indicating that NL2 immunoreactivity is predictive of the location of inhibitory synapses within this brain region. In tissue from WT mice, NL2 immunoreactivity was predictably enriched within *stratum pyramidale* with lower levels of immunoreactivity evident in *strata oriens, radiatum and lacunosum moleculare* (Fig. 1 A1). In *stratum radiatum*, a distinct gradient was noticeable with most of the signal concentrated within the proximal regions of this layer, decreasing to negligible amounts in the distal regions. This varying parcelation of NL2 immunoreactivity into distinct layers closely follows the distribution of inhibitory synapses within this brain region [30]. High resolution inspection of NL2 immunoreactivity confirmed that within *stratum pyramidale,* the signal presented as individual clusters located on the plasma membranes of somata and proximal apical dendritic profiles, delineated by Kv2.1 immunoreactivity (Fig. 1 A2, 3). In tissue from mdx mice reacted and imaged under conditions identical to those used for the WT tissue, the characteristic enrichment of NL2 labelling pattern within *stratum pyramidale* evident in the WT tissue was less striking. Instead, diffuse signal within this layer closely matched the intensity of signal in *stratum radiatum*. In contrast, the density of NL2 with *stratum lacunosum moleculare* appeared similar to levels in the tissue from WT (Fig. 1 B). This suggests that the influence of dystrophin on the location of NL2 immunoreactivity is specific to *strata pyramidale* and *radiatum*. The apparent redistribution of NL2 immunoreactivity within the CA1 sub-field in dystrophin-deficient mice was most pronounced in tissue from our mdx^βgeo^ mouse model (lacking all dystrophins) where no apparent gradient in labelling intensity between *strata* was evident (Fig. 1 C). Quantification of NL2 immunoreactivity revealed a significant decrease in the density of NL2 immunoreactive clusters within *stratum pyramidale* of dystrophin-deficient mice compared to WT mice (mean ± SEM; WT 15±1 clusters per 100 µm^2^, mdx 10±1 clusters per 100 µm^2^, mdx^βgeo^ 10±1 clusters per 100 µm^2^; P<0.0001, ANOVA, N = 3 animals per genotype) (Fig. 1 D1) with a significant increase in density evident within the distal *stratum radiatum* (mean ± SEM; WT 5±1 clusters per 100 µm^2^, mdx 7±1 clusters per 100 µm^2^, mdx^βgeo^ 9±1 clusters per 100 µm^2^; P<0.0001, ANOVA, N = 3 animals per genotype) (Fig. 1 D2). Collectively, the data indicate that the deletion of dystrophin impacts on the location of NL2 expression which suggests the formation of misplaced inhibitory synapses within the CA1 region of the mouse hippocampus.

### Immunoreactivity for the GABAergic axonal terminal marker protein vesicular GABA transporter (VGAT) mirrors the changes in NL2 expression in the absence of dystrophin

**Figure 2:**
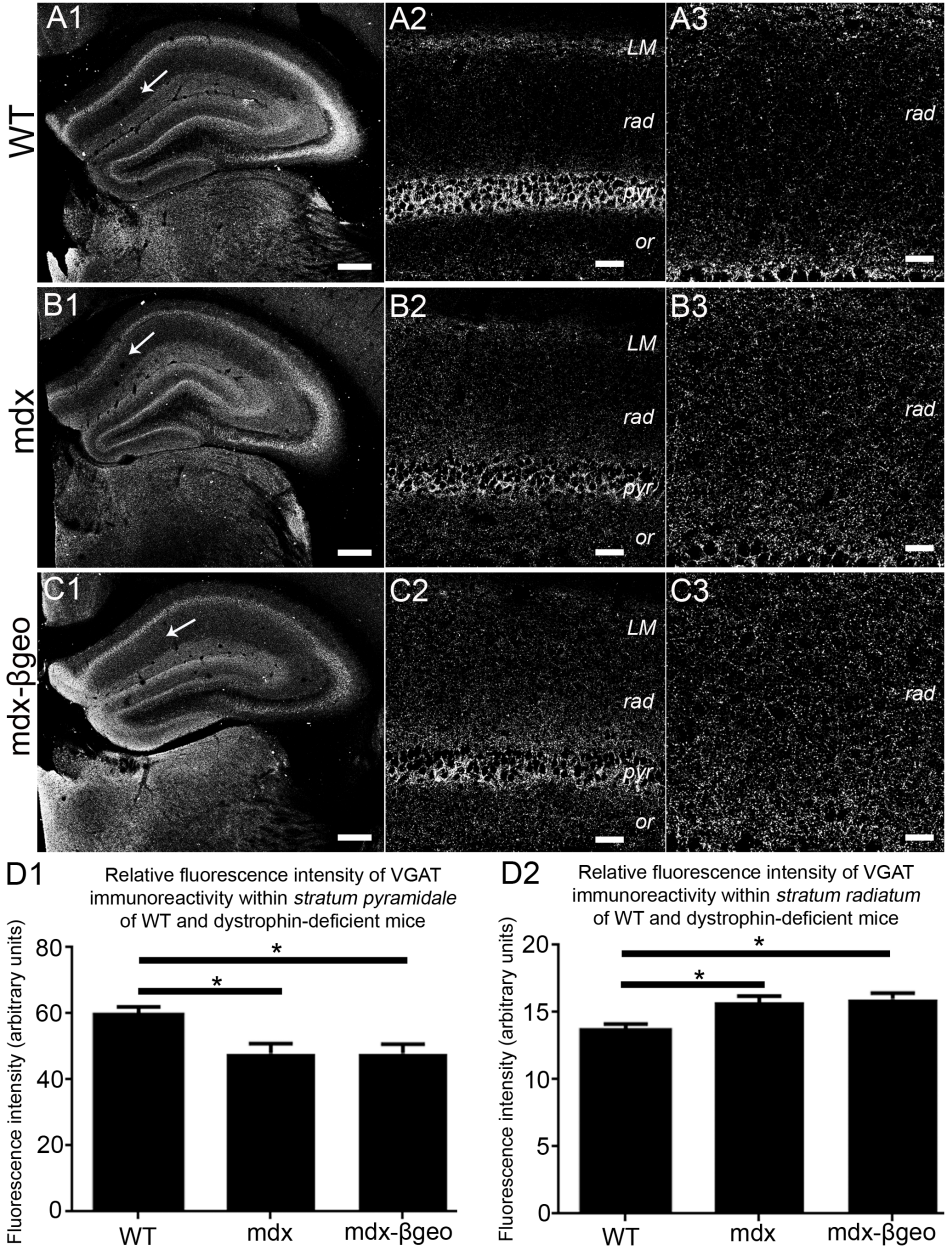
Localisation and quantification of VGAT immunoreactivity within the hippocampus of WT and dystrophin-deficient mice, reacted and imaged under identical conditions. (A) shows the pattern of VGAT immunoreactivity within the hippocampus of WT mouse tissue. (A 1) an overview of the entire hippocampus demonstrating the prototypical enrichment of VGAT immunoreactivity within the cell body regions of the hippocampus. (A 2) a magnified view of the CA1 sub-field confirming the enrichment of VGAT immunoreactivity in *stratum pyramidale* compared with the dendritic regions. (A 3) is magnified view of *stratum radiatum* showing the comparatively sparse level of VGAT immunoreactivity within this layer in WT mice. (B) and (C) show the pattern of VGAT immunoreactivity within the hippocampus of mdx and mdx^βgeo^ mouse tissue respectively, reacted and imaged under conditions identical to those for WT tissue. Whilst the enrichment within (B 2, C 2) *stratum pyramidale* is still evident, the level of signal is significantly lower compared to WT. There is also a noticeably higher level VGAT immunoreactivity within (B 3, C 3) *stratum radiatum* compared to WT. (D 1) quantification of the intensity of VGAT immunoreactivity within *stratum pyramidale* of WT, mdx and mdx^βgeo^ tissue. (D 2) quantification of the intensity of VGAT immunoreactivity in the *stratum radiatum* of WT, mdx and mdx^βgeo^ tissue. Bars represent the means and lines the SEM. *P<0.05; ANOVA with Tukey’s posthoc test; N = 3 animals. Scale bars (A1, B1, C1) 200 µm; (A2, B2, C2) 40 µm; (A3, B3, C3) 20 µm.

We exploited the immunoreactivity pattern of the vesicular GABA transporter (VGAT) to investigate whether the distribution of GABAergic innervation in the CA1 sub-field of WT and dystrophin-deficient mice mirrored the altered pattern of putative inhibitory synapses inferred by the changed NL2 immunoreactivity. In tissue from WT mice, predictably, VGAT immunoreactivity was enriched in *strata pyramidale* and *lacunosum moleculare* with a significantly lower level of expression in *strata oriens* and *radiatum* (Fig. 2 A 1–3). In tissue from mdx mice, VGAT signal was still concentrated in *strata pyramidale* and *lacunosum moleculare* compared to other layers, although it was at lower levels when compared to the signal in WT tissue (Fig. 2 B1). However, higher levels of VGAT immunoreactivity were evident in *stratum radiatum* of mdx tissue when compared to the level of signal within this layer of the WT tissue (Fig. 2 B2, 3). This trend towards a relatively even distribution of VGAT immunoreactivity across *strata pyramidale* and *radiatum* was most striking in tissue from mdx^βgeo^ mice (Fig. 2 C). Quantification of VGAT immunofluorescence intensity in *stratum pyramidale* confirmed a significant decrease in the intensity of the signal in mdx and mdx^βgeo^ mice compared to WT mice (mean ± SEM in arbitrary units, AU; WT 60±2 AU, mdx 48±3 AU, mdx^βgeo^ 48±2 AU; P<0.0001, ANOVA, N = 3 animals per genotype) (Fig. 2 D1). In contrast, there was a significant increase in the intensity of VGAT immunoreactivity signal in *stratum radiatum* of mdx and mdx^βgeo^ mice compared to WT mice (mean ± SEM in arbitrary units, AU; WT 14±1 AU, mdx 16±1 AU, mdx^βgeo^ 16±1 AU; P<0.0001, ANOVA, N = 3 animals per genotype) (Fig. 2 D2). Collectively, the redistribution of NL2 and VGAT immunoreactivity from *strata pyramidale* to *radiatum* in mdx and mdx^βgeo^ mice suggests that dystrophin is associated with the patterning of GABAergic innervation in the mouse CA1 sub-field. Thus, dystrophin appears to be essential for the stereotypical expression patterning of both pre- and postsynaptic proteins associated with GABAergic transmission within the CA1 region of the hippocampus. If so, this is likely to manifest in an alteration in the location of putative GABA release sites within the region. To verify this, we examined the well-described axonal targeting patterns of interneurons, which provide the major source of GABA within this region.

### The absence of dystrophin alters the location of proteins located within the axons of specific classes of interneurons

**Figure 3:**
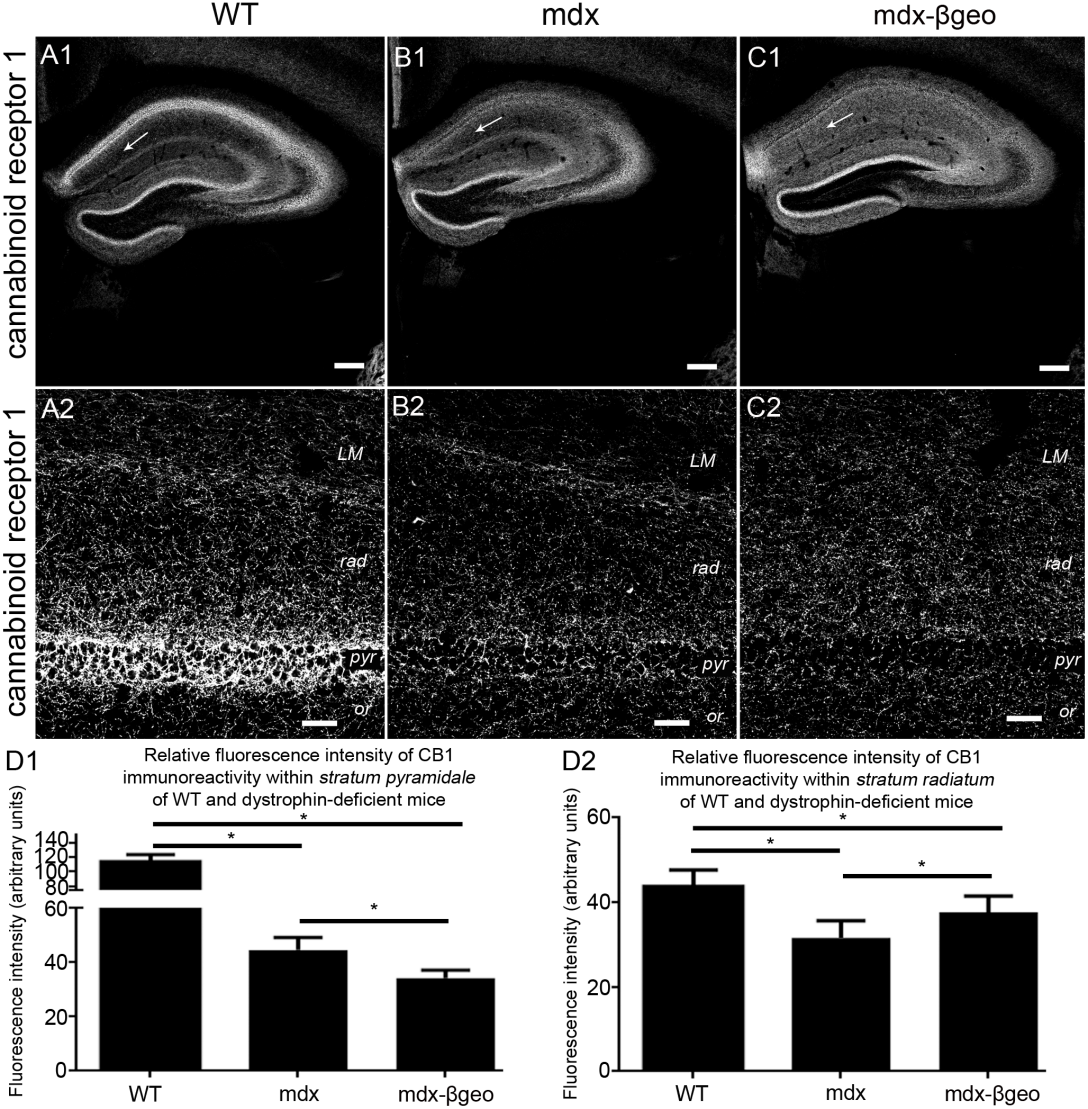
Localisation and quantification of CB1 immunoreactivity within the hippocampus of WT and dystrophin-deficient mice, reacted and imaged under identical conditions. (A) shows the pattern of CB1 immunoreactivity within the hippocampus of WT mouse tissue. (A 1) an overview of the entire hippocampus demonstrating the prototypical enrichment of VGAT immunoreactivity within the cell body regions of the hippocampus compared to one of the dendritic regions (arrow). (A 2) a magnified view of the CA1 sub-field confirming the enrichment of CB1 immunoreactivity in *stratum pyramidale* compared with the dendritic regions. (B) and (C) show the pattern of CB1 immunoreactivity within the hippocampus of mdx and mdx^βgeo^ mouse tissue respectively, reacted and imaged under conditions identical to those for the WT tissue. Note the significant level of signal within the dendritic layers, particularly in mdx^βgeo^ mouse tissue (arrow). Whilst the enrichment within (B 2, C 2) *stratum pyramidale* is still evident, the level of signal is significantly lower compared to WT. (D 1) quantification of the intensity of CB1 immunoreactivity within *stratum pyramidale* of WT, mdx and mdx^βgeo^ tissue. (D 2) quantification of the intensity of CB1 immunoreactivity in the *stratum radiatum* of WT, mdx and mdx^βgeo^ tissue. Bars represent the means and lines the SEM. *P<0.05; ANOVA with Tukey’s posthoc test; N = 3 animals. Scale bars (A1, B1, C1) 200 µm; (A2, B2, C2) 40 µm.

Numerous functionally and neurochemically distinct types of interneurons preferentially innervate specific sub-cellular domains of CA1 pyramidal cells [21], [35]. Since the above data suggest an apparent redistribution of inhibitory pre- and postsynaptic molecular markers between *strata pyramidale* and *radiatum*, we investigated whether this altered pattern of inhibitory innervation may be reflected within the axonal projections of specific classes of interneuron. Axon terminals innervating the perisomatic domains of CA1 pyramidal neurons arise exclusively from two broad classes of basket cells identified by their expression of either the calcium binding protein parvalbumin (PV) [36] or the neuropeptide cholecystokinin (CCK) [37]. The majority of CCK-expressing basket cells also express the cannabinoid receptor 1 (CB1) in their axon terminals [38]. We therefore investigated the distribution of CB1 immunoreactivity within the CA1 sub-field. CB1 signal was characteristically enriched within *stratum pyramidale* of the tissue from WT mice (Fig. 3 A1) with significantly lower levels in *stratum radiatum* (Fig. 3 A2). In comparison, there was a noticeable decrease in the intensity of CB1 immunoreactivity in *stratum pyramidale* of this tissue from mdx mice, reacted and imaged under conditions identical to those used for WT samples (Fig. 3 B1), although there was still an apparent gradient in the signal within *stratum radiatum* (Fig. 3 B2). The dramatic decrease in the intensity of CB1 immunoreactivity in *stratum pyramidale* was most pronounced in tissue from mdx^βgeo^ mice (Fig. 3 C1), in which, comparatively more intense CB1 signal was evident in *stratum radiatum* (Fig. 3 C2). Quantification of the intensity of CB1 immunoreactivity confirmed a significant decrease in the levels of CB1 staining in the *stratum pyramidale* of dystrophin-deficient mice (mean ± SEM in arbitrary units, AU; WT 114±9 AU, mdx 44±5 AU, mdx^βgeo^ 34±3 AU; P<0.0001, ANOVA, N = 3 animals per genotype) (Fig. 3 D1). Interestingly, while in WT mice, the intensity of staining in *stratum radiatum* was predictably less than that in *stratum pyramidale,* signal within the *stratum radiatum* of mdx^βgeo^ was significantly more than that in the *stratum pyramidale* of this genotype, but still significantly less than that in WT (mean ± SEM in arbitrary units, AU; WT 44±3 AU, mdx 32±4 AU, mdx^βgeo^ 38±3 AU; P<0.0001, ANOVA, N = 3 animals per genotype) (Fig. 3 D2).

**Figure 4:**
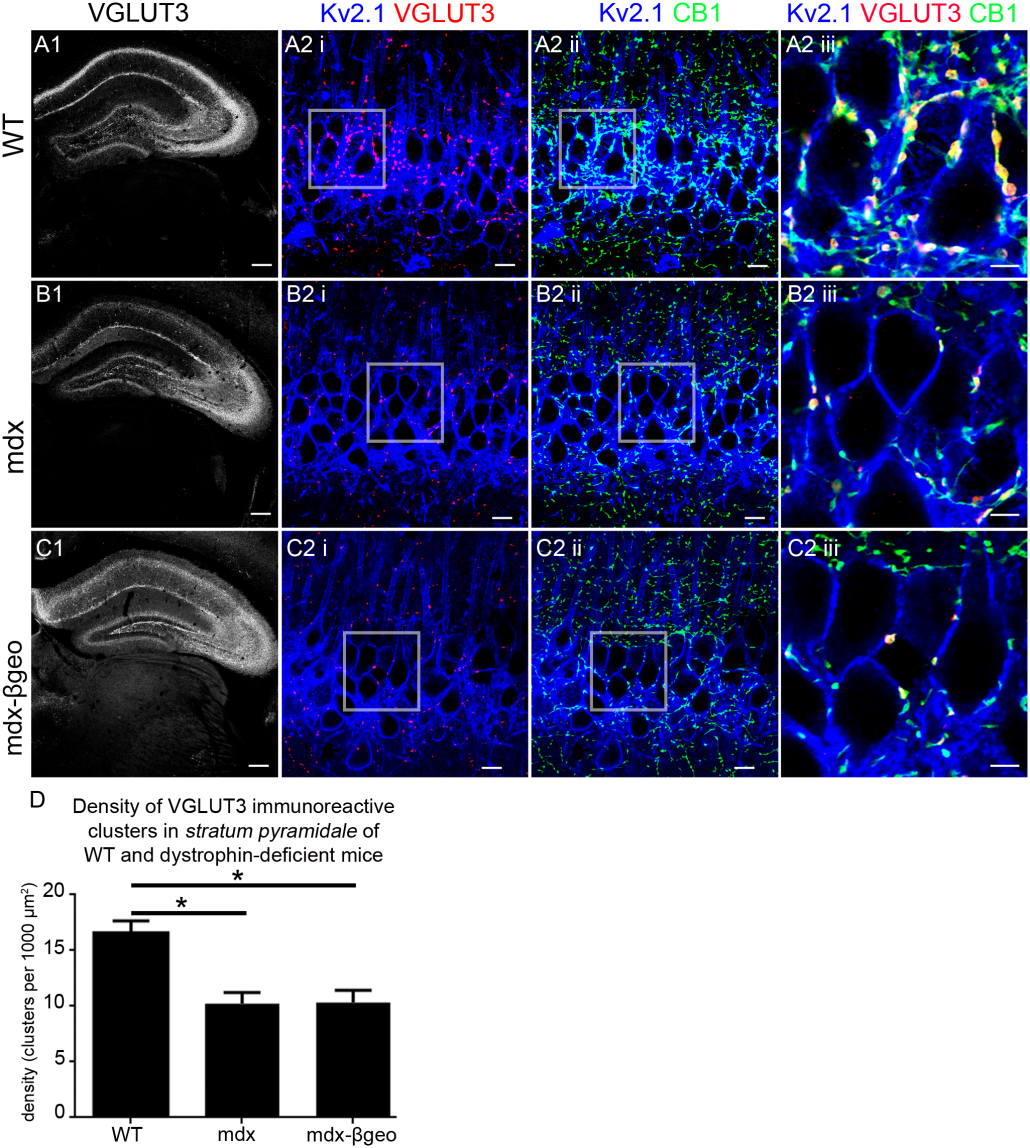
Localisation and quantification of VGLUT3 immunoreactivity within the hippocampus of WT and dystrophin-deficient mice, reacted and imaged under identical conditions. (A) shows the pattern of VGLUT3 immunoreactivity within the hippocampus of WT mouse tissue. (A 1) an overview of the entire hippocampus demonstrating the prototypical enrichment of VGLUT3 immunoreactivity within the cell body regions of the hippocampus with lower levels evident in distal dendritic layers. (A 2 i) and (A 2 ii) shows puncta immunoreactive for VGLUT3 and CB1 respectively on Kv2.1 immunopositive somato-dendritic profiles within *stratum pyramidale*. (A 2 iii) shows that virtually all VGLUT3 immunopositive puncta colocalise with those immunopositive for CB1 and are thus likely to represent the axon terminals of cholecystokinin-expressing basket cells. (B) and (C) show the pattern of VGLUT3 immunoreactivity within the hippocampus of mdx and mdx^βgeo^ mouse tissue respectively, reacted and imaged under conditions identical to those for the WT tissue. (B 2 I, ii) and (C 2 I, ii) demonstrate the significantly lower levels of VGLUT3 and CB1 immunoreactivity within the *stratum pyramidale* of mdx and mdx^βgeo^ mouse tissue respectively. (B 2 iii) and (C 2 iii) demonstrate the sparse number of colocalised VGLUT3 and CB1 immunoreactive clusters suggestive of a significant decrease in the density of innervation by cholecystokinin-expressing basket cells within this region of dystrophin-deficient mice. (D) quantification of the density of VGLUT3 immunoreactive clusters in *stratum pyramidale* of WT, mdx and mdx^βgeo^ tissue. Bars represent the means and lines the SEM. *P<0.05; ANOVA with Tukey’s posthoc test; N = 3 animals. Scale bars (A, B, C, 1) 200 µm; (A, B, C, 2 i, ii) 10 µm; (A, B, C, 2 iii) 5 µm.

We next used an additional marker of CCK-expressing basket cell axon terminals, namely the vesicular glutamate transporter 3 (VGLUT3) [39], in order to verify the potential changes in this population of axonal projections in the absence of dystrophin. In tissue from WT mice, VGLUT3 immunoreactivity was characteristically enriched within *stratum pyramidale* and to a lesser degree in *stratum lacunosum moleculare* (Fig. 4 A1). High resolution visualisation revealed that within *stratum pyramidale*, individual VGLUT3 immunoreactive clusters contacted the somatic and proximal dendritic compartments of KV2.1 immunopositive neurons (Fig. 4 A2i). Co-labelling with CB1 (Fig. 4 A2iii) showed complete colocalisation with VGLUT3 immunopositive clusters (Fig. 4 A2iii) indicating that these VGLUT3 immunopositive clusters are likely to represent axon terminals of CCK-expressing basket cells. In concordance with CB1 and VGAT immunoreactivity profiles, there was a striking decrease in the intensity of the VGLUT3 signal in the *stratum pyramidale* of mdx tissue and to a lesser extent in *stratum lacunosum moleculare* (Fig. 4 B1). In comparison to WT tissue, sparse VGLUT3 immunoreactive clusters contacted Kv2.1 immunoreactive somata and dendrites in *stratum pyramidale* and colocalised with CB1 immunoreactive puncta (Fig. 4 B2). This decreased intensity in VGLUT3 immunoreactivity in the absence of dystrophin was mirrored in tissue from mdx^βgeo^ mice (Fig. 4 C). Quantification of the density of VGLUT3 immunoreactive clusters within the *stratum pyramidale* confirmed the significant decrease in dystrophin-deficient mice compared to WT (mean ± SEM; WT 17±1 clusters per 1000 µm^2^, mdx 10±1 clusters per 1000 µm^2^, mdx^βgeo^ 10±1 clusters per 1000 µm^2^; P<0.0001, ANOVA, N = 3 animals per genotype) (Fig. 4 D). The implication is that dystrophin expression within CA1 pyramidal neurons is essential for the targeting of CCK-expressing basket cell axons to the precise perisomatic domains of pyramidal neurons.

**Figure 5:**
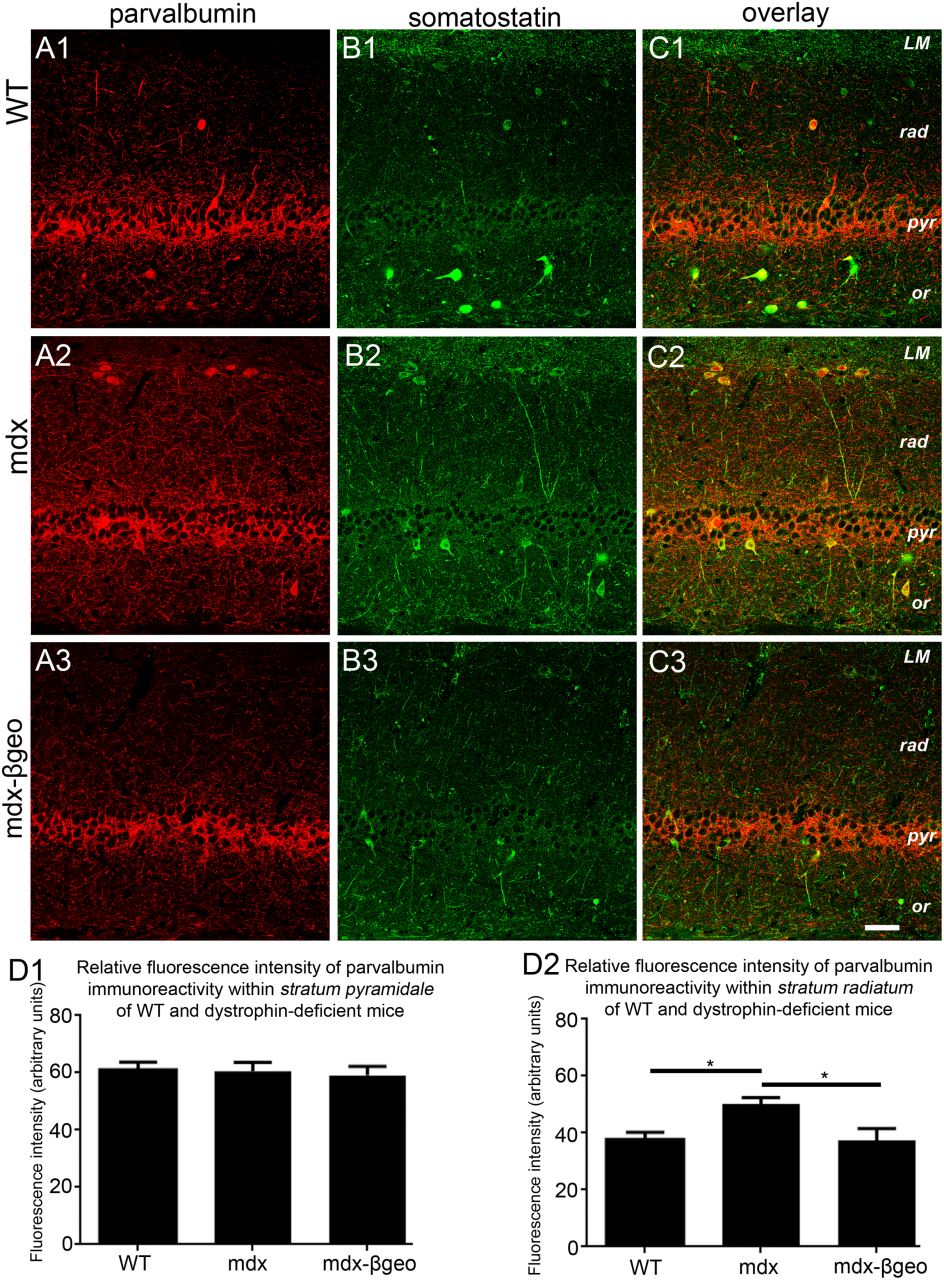
Localisation and quantification of PV and somatostatin immunoreactivity within the hippocampus of WT and dystrophin-deficient mice, reacted and imaged under identical conditions. (A 1), (A 2) and (A 3) show PV immunoreactivity within the CA1 region of WT, mdx and mdx^βgeo^ tissue respectively. Note the significant increase in the level of PV immunoreactivity within *stratum radiatum* of mdx tissue. (B 1), (B 2) and (B 3) show somatostatin immunoreactivity within the CA1 region of WT, mdx and mdx^βgeo^ tissue respectively. Note the significantly increase in the level of somatostatin immunoreactivity particularly within *stratum radiatum* of mdx tissue. Images in (C) are overlays of (A) and (B). Note the significant number of PV-somatostatin co-expressing neurons in (C 2) tissue from mdx mice. (D 1) quantification of the intensity of PV immunoreactivity within *stratum pyramidale* of WT, mdx and mdx^βgeo^ tissue. (D 2) quantification of the intensity of PV immunoreactivity in the *stratum radiatum* of WT, mdx and mdx^βgeo^ tissue. Bars represent the means and lines the SEM. *P<0.05; ANOVA with Tukey’s posthoc test; N = 3 animals. Scale bar 50 µm.

Apart from CCK-expressing basket cells, parvalbumin (PV)-containing basket cells provide the remaining inhibitory input onto somatic and proximal dendritic domains of CA1 pyramidal cells. We therefore explored the pattern of PV immunoreactivity in the CA1 sub-field of WT and dystrophin-deficient mice. In tissue from WT mice, PV immunoreactivity was characteristically concentrated in axonal profiles preferentially located in *stratum pyramidale* with signal also evident in isolated somata located in *strata oriens and radiatum* (Fig. 5 A1). In tissue from mdx mice, there was no discernible difference in the intensity of PV immunoreactivity within *stratum pyramidale.* However, PV immunoreactivity within dendritic layers such as *strata oriens* and *radiatum* appeared elevated compared to these corresponding layers in WT tissue (Fig. 5 A2). In contrast, PV immunoreactivity profile within tissue from mdx^βgeo^ mice appeared indistinct to that of WT tissue (Fig. 5 A3).

Intriguingly, an increase in the number of PV-expressing neurons has been reported in the hippocampus of mdx mice [40], however, it is not clear which particular sub-population of PV-expressing interneurons is reflected in this increased density. In the hippocampus, interneurons termed bistratified cells express both PV and somatostatin, and project their axons preferentially to dendritic regions [41], that is the location of elevated PV signal in mdx mice. We therefore investigated the pattern of somatostatin immunoreactivity in order to elucidate the possible source of the more widespread PV innervation within dendritic layers. In tissue from WT mice, somatic immunoreactivity for somatostatin was evident primarily in *strata oriens* and *pyramidale* with immunoreactive varicosities distributed throughout *strata oriens, radiatum and lacunosum moleculare* (Fig. 5 B1). In mdx tissue, the pattern of somatostatin signal was markedly increased compared to WT with a higher level of immunoreactivity across dendritic layers of the CA1 sub-field (Fig. 5 B2). Somatostatin immunoreactivity in tissue from mdx^βgeo^ mice appeared less intense than that in mdx tissue but was also evenly distributed across CA1 *strata* (Fig. 5 B3). Putative bistratified interneurons, identified by their co-expression of both PV and somatostatin, were evident in all genotypes, but were particularly noticeable in tissue from mdx mice (Fig. 5 C1–3).

Quantification of the intensity of PV immunoreactivity revealed that within *stratum pyramidale*, there were no significant differences across genotypes (mean ± SEM in arbitrary units, AU; WT 61±3 AU, mdx 61±3 AU, mdx^βgeo^ 60±3 AU; P = 0.4, ANOVA, N = 3 animals per genotype) (Fig. 5 D1). However, within *stratum radiatum*, the intensity of PV immunoreactivity in tissue from mdx mice was significantly higher than that of WT and mdx^βgeo^ mice (mean ± SEM in arbitrary units, AU; WT 38±3 AU, mdx 50±2 AU, mdx^βgeo^ 37±4 AU; P<0.0001, ANOVA, N = 3 animals per genotype) (Fig. 5 D2). Collectively, these data suggest more widespread innervation in dystrophin-deficient mice by those PV-containing interneurons, which preferentially target dendritic domains of pyramidal neurons; these are likely to be PV-somatostatin co-expressing bistratified neurons. To assess whether the increased levels of immunoreactivity of these neurochemicals (PV, somatostatin) within mdx mice might be a manifestation of either increased PV, somatostatin, or PV-somatostatin (that is, bistratified cells) cell density, we counted the number of PV, somatostatin and PV-somatostatin expressing neurons within strata *oriens*, *pyramidale* and *radiatum* in WT, mdx and mdx^βgeo^ mice. We detected a significant increase the number of PV, somatostatin and PV-somatostatin expressing neurons in tissue from mdx mice throughout all layers of the CA1 region whilst higher numbers of such immunopositive neurons were also evident in the *stratum radiatum* of mdx^βgeo^ tissue, when compared with tissue from WT mice (Table 2). Collectively, the data suggest that the absence of dystrophin profoundly affects the expression pattern of somatostatin within this brain region. It should be noted that within this brain region, somatostatin is also expressed by O-LM cells [42], [43] and hippocamposeptal neurons [44] but these cells do not co-express PV. Functional studies in which the recorded cells are labelled and unequivocally identified [43], [45] would thus be required to determine which particular interneuron-type exhibits altered projection patterns in the absence of dystrophin.

**Table 2:** Quantification of the number of PV and somatostatin immunopositive cells within the various layers of the CA1 sub-field of WT and dystrophin-deficient mice per 60 µm-thick tissue section.

	Stratum oriens			Stratum pyramidale			Stratum radiatum		
	WT	mdx	mdx^βgeo^	WT	mdx	mdx^βgeo^	WT	mdx	mdx^βgeo^
parvalbumin	22±3	33±3	21±3	30±3	44±3	35±4	19±3	70±4	33±8
somatostatin	20±4	42±3	26±5	21±3	33±4	35±5	18±4	34±5	16±2
parvalbumin- somatostatin colocalisation	7±1	21±2	10±2	13±2	22±3	24±3	16±4	24±4	11±1

Values represent the mean ± SD values per tissue section, 3–5 tissues per animal, 3 animals per genotype.

### Altered distribution and synaptic clustering of the GABA-A receptor (GABA_A_R) alpha1 subunit in dystrophin-deficient mice

**Figure 6:**
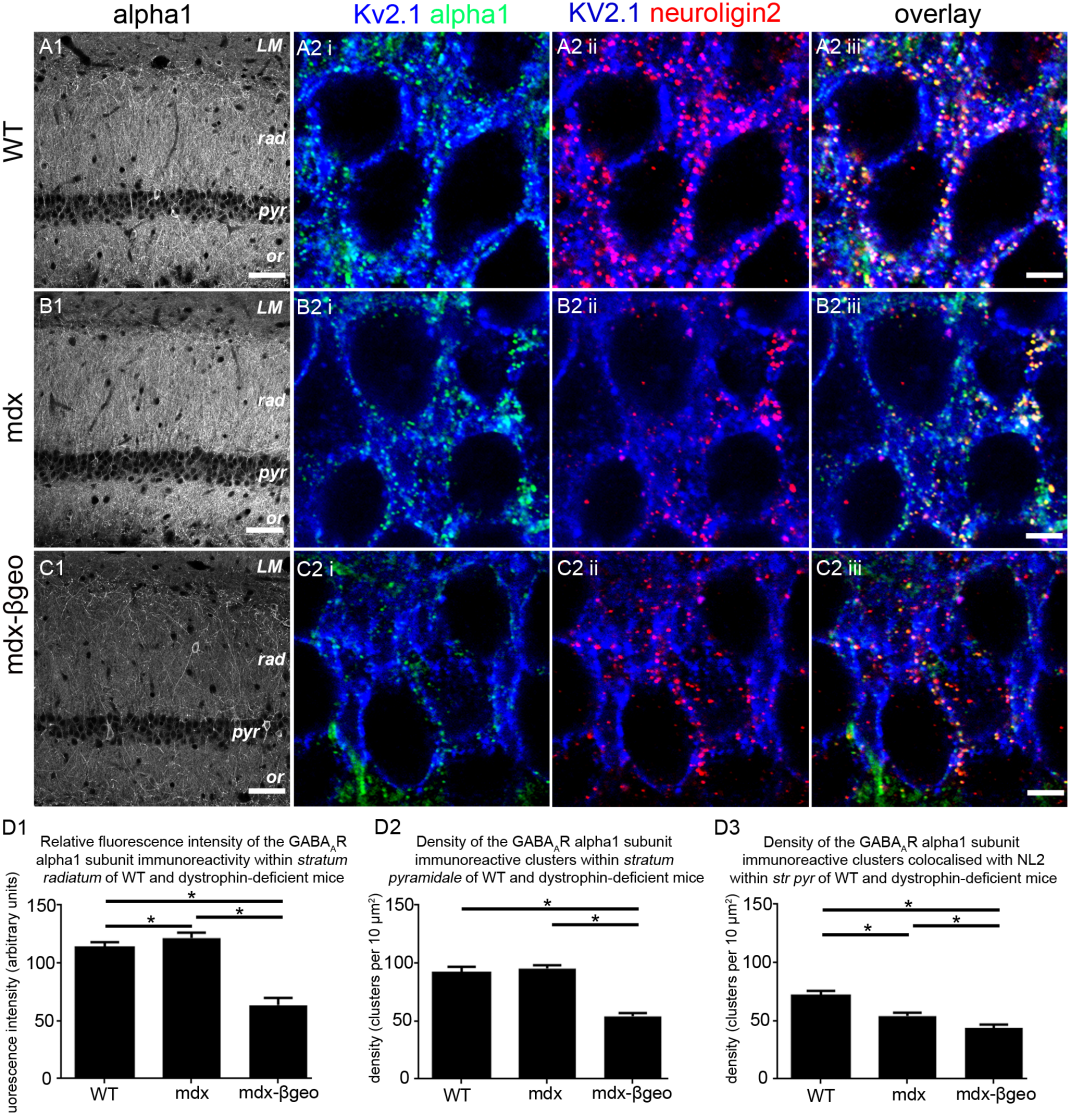
Localisation and quantification of GABA_A_R alpha1 subunit immunoreactivity within the CA1 region of the hippocampus of WT and dystrophin-deficient mice, reacted and imaged under identical conditions. (A 1) shows that, in WT tissue, alpha1 subunit immunoreactivity is enriched within the dendritic layers of *strata oriens* and *radiatum* with comparatively lower levels evident in *strata pyramidale* and *lacunosum moleculare*. (A 2) shows that within *stratum pyramidale*, the majority of alpha1 subunit immunoreactive clusters colocalise with clusters immunopositive for NL2 on Kv2.1 somato-dendritic cell surfaces. (B 1) shows that within the CA1 region of mdx mice, there is an apparent increase in the intensity of alpha1 subunit immunoreactivity within *strata oriens* and *radiatum* when compared to WT. (B 2) shows that within *stratum pyramidale*, there is comparatively less colocalisation between alpha1 subunit and NL2 immunopositive clusters. (C 1) shows that within the CA1 region of mdx^βgeo^ mice, there is a striking decrease in the intensity of alpha1 subunit immunoreactivity throughout all layers when compared to WT. Accordingly, (C 2) shows that there is significantly less colocalisation between alpha1 subunit and NL2 immunopositive clusters when compared to WT. (D 1) quantification of the intensity of alpha1 subunit immunoreactivity within *stratum radiatum* of WT, mdx and mdx^βgeo^ tissue. (D 2) quantification of the density of alpha1 subunit immunoreactive clusters in *stratum pyramidale* of WT, mdx and mdx^βgeo^ tissue. (D 3) quantification of the density of alpha1 subunit immunoreactive clusters colocalised with clusters immunoreactive for NL2 in *stratum pyramidale* of WT, mdx and mdx^βgeo^ tissue. Bars represent the means and lines the SEM. *P<0.05; ANOVA with Tukey’s posthoc test; N = 3 animals. Scale bars (A, B, C, 1) 50 µm; (A, B, C, 2) 5 µm.

The data above indicate alterations in the stereotypical projection patterns of GABAergic axons in the CA1 sub-field. If so, this altered release of GABA within different *strata* is likely to require a parallel change in the expression pattern of GABA receptors required to mediate the effects of this neurotransmitter within this region. To examine this, we focused on GABA_A_Rs, and in particular, the most abundant subtype in the brain, namely those containing the alpha1 subunit [46]. The distribution of GABA_A_R alpha1 subunit immunoreactivity in the CA1 region of the WT mice was similar to that previously reported [47], [48], being concentrated in *strata oriens and radiatum* with lower levels in *stratum pyramidale* (Fig. 6 A1). The level of GABA_A_R alpha1 subunit immunoreactivity was noticeably higher in *strata oriens and radiatum* of mdx tissue (Fig. 6 B1). However, in stark contrast, the level of GABA_A_R alpha1 subunit immunoreactivity in mdx^βgeo^ mice was significantly lower than that of WT and mdx across all layers (Fig. 6 C1). Quantification of the intensity of GABA_A_R alpha1 subunit immunoreactivity within *stratum radiatum* confirmed a significant increase in mdx tissue whilst the levels in tissue from mdx^βgeo^ mice was significantly lower, compared to WT tissue (mean ± SEM in arbitrary units, AU; WT 113±2 AU, mdx 122±2 AU, mdx^βgeo^ 64±2 AU; P<0.0001, ANOVA, N = 3 animals per genotype) (Fig. 6 D1).

These divergent levels of GABA_A_R alpha1 subunit immunoreactivity within the dendritic layers of the CA1 region were accompanied by altered levels of colocalisation with NL2 immunopositive clusters within *stratum pyramidale*. In tissue from WT mice, virtually all NL2 immunoreactive clusters co-localised with those of the GABA_A_R alpha1 subunit in *stratum pyramidale* suggesting that the majority of inhibitory synapses within this layer, presumably located on pyramidal somata, expressed alpha1 subunit-containing GABA_A_Rs (Fig. 6 A2), in agreement with previously published evidence [49]. However, in tissue from mdx and mdx^βgeo^ mice, only sparse co-localisation between NL2 and GABA_A_R alpha1 subunit immunoreactive clusters was evident (Fig. 6 B2, C2). A reason for the low degree of GABA_A_R alpha1 subunit colocalisation with NL2 could be the significant decrease in the density of NL2 immunoreactive clusters in dystrophin-deficient mice (Fig. 1) or a decrease in the density of GABA_A_R alpha1 subunit expression. To determine this, we quantified the density of GABA_A_R alpha1 subunit immunoreactive clusters, as well as the density of NL2- GABA_A_R alpha1 subunit colocalised clusters. There were no significant differences in the density of GABA_A_R alpha1 subunit immunoreactive clusters within *stratum pyramidale* of WT and mdx mice, although the density within mdx^βgeo^ was significantly lower than both WT and mdx (mean ± SEM; WT 92±2 clusters per 100 µm^2^, mdx 95±1 clusters per 100 µm^2^, mdx^βgeo^ 54±1 clusters per 100 µm^2^; P<0.0001, ANOVA, N = 3 animals per genotype) (Fig. 4 d2). However, there was a significant decrease in the density of NL2- GABA_A_R alpha1 subunit colocalised clusters in both mdx and mdx^βgeo^ tissue (mean ± SEM; WT 72±2 clusters per 100 µm^2^, mdx 54±1 clusters per 100 µm^2^, mdx^βgeo^ 44±1 clusters per 100 µm^2^; P<0.0001, ANOVA, N = 3 animals per genotype) (Fig. 4 D2). Collectively, the data suggest changes in the patterns of GABA_A_R alpha1 subunit immunoreactivity which mirror those of inhibitory synaptic marker proteins within this brain region.

## Discussion

The current study shows that the mouse models in which dystrophin is constitutively deleted present with a complex pattern of alterations in the location of proteins associated with both the pre- and postsynaptic elements of inhibitory synapses as well as those proteins located in the axon terminals of specific GABAergic interneuron classes. The data suggest that in the absence of dystrophin, there is a rearrangement of the molecular machinery which underlies the precise spatio-temporal pattern of GABAergic synaptic transmission within the CA1 sub-field of the hippocampus. This could lead to an imbalance in the exquisite choreography of cellular network activity that underlies salient aspects of hippocampal function such as learning and memory, thereby contributing to the cognitive impairment associated with dystrophin-mutations leading to DMD.

### Alterations in the density and location of pre- and post-synaptic inhibitory marker proteins

Coordinated brain activity relies on the rhythmic activity of functionally distinct cell-types operating within strict spatio-temporal constraints [43]. Central to such optimal cellular communication, and thus coordinated neuronal activity between ensembles of neuronal networks, is the precise patterning of inhibitory and excitatory synaptic connections between neuronal classes [50], [51]. The relationship between the type of synapse and their sub-cellular location has been best characterised in the CA1 sub-field of the hippocampus [30]. CA1 pyramidal neurons elaborate several morphologically distinct dendritic arbours onto which excitatory and inhibitory synapses are targeted to specific sub-cellular domains, allowing for the spatial segregation of information flow from diverse cell-types; see [21], [35] for review. This compartmentalisation of functionally distinct synaptic inputs onto precise sub-cellular domains of individual pyramidal neurons allows for the integration of excitatory and inhibitory synaptic transmission. Therefore, the altered distribution of NL2 immunoreactivity, and presumably inhibitory synapses, within the CA1 region of the hippocampus in dystrophin-deficient mice suggests a role for dystrophin in the correct patterning of GABAergic synapses on specific sub-cellular domains of CA1 pyramidal neurons. Its precise role is unclear but is likely to involve pre-and postsynaptic signalling since we show mirrored changes in the projection patterns of neurochemicals which are expressed by defined classes of interneurons. NLs, together with neurexins, are transynaptic adhesion molecules [52], [53]. NLs are required for correct synapse maturation but not for the initial formation of synaptic contacts [33]. Since dystroglycan, a member of the DAP complex which is required for the localisation of dystrophin to GABA_A_R clusters, is also the physiological ligand for neurexins [19] it is conceivable that the absence of dystrophin impairs the ability of NLs and neurexins to initiate the maturation of appropriate pre- and postsynaptic membranes amongst the many redundant immature synapses formed during development. This potential role of dystrophin in synapse maturation appears to be restricted to specific cell-types. For example, CB1-VGLUT3-expressing varicosities which arise predominantly from the CCK-expressing basket cells were distributed relatively evenly across dendritic fields in dystrophin-deficient mice (Figures 3, 4) as opposed to their characteristic enrichment within the cell body layer in WT mice [38]. Intriguingly, there were no apparent changes in the level of immunoreactivity for PV-expressing varicosities within *stratum pyramidale* although increased PV signal, together with that of somatostatin, was evident in dendritic layers. Such increased PV-somatostatin expression could represent alterations in bistratified cells, a class of interneuron which preferentially targets the dendritic domains of pyramidal neurons. However, the precise mechanisms which underlie this cell-type selectivity of dystrophin in influencing interneuron projection patterns are currently unclear. Likely factors could be the developmental onset of dystrophin expression within the hippocampus, the developmental origins of various interneuron types and the time course of hippocampal invasion by such cells. Dystrophin expression within the mouse CNS commences from embryonic day 14 onwards [54] which closely correlates with the onset of interneuron invasion of the hippocampus [55]. However, CCK-expressing cells are generated later and from the caudal ganglionic eminence compared to PV-expressing cells which originate from the medial ganglionic eminence [55]. A further factor is likely to be the initial pattern of synapses formed during development. CCK-expressing basket cells, prior to providing inhibitory input onto pyramidal cells somata in adulthood, initially form synapses on pyramidal cell dendrites during early postnatal stages where they are postulated to provide GABA-mediated depolarising input to augment immature glutamatergic synaptic transmission [56]. It is currently unclear whether such initial dendritic innervation occurs with PV-expressing basket cells due to the low levels of PV expression during development [57], [58]. Nevertheless, it is tempting to speculate that the absence of dystrophin impacts on the relocation of these initial synaptic contacts from dendritic compartments to their eventual somatic locations, as evidenced by the paucity of NL2 immunoreactivity in the pyramidal cell body layer of dystrophin-deficient mouse models together with abnormal levels of expression in dendritic layers. As such, this suggests a seminal role for dystrophin in the proper functioning of NL-neurexin synapse maturation pathways.

### Alterations in the projection patterns of interneurons

Functionally distinct classes of interneurons shape the patterns of activity of principal neurons within the cortex [21], [35]. They achieve this by releasing GABA to precise sub-cellular domains of the principal cells in defined amounts at appropriate times in a brain state [43] and behaviour-dependent manner [59]. Therefore, an imbalance to the rhythmicity orchestrated by the different classes of interneurons within the brain region is likely to be the functional consequences of the anatomical data showing an altered distribution of VGAT, CB1, VGLUT3 and somatostatin-expressing varicosities in the CA1 sub-field of dystrophin-deficient mice. For example, PV and CCK-expressing basket cells both provide inhibitory input on the somata of hippocampal pyramidal neurons yet occupy distinct roles in terms of different hippocampal network oscillations [60]. Since CB1-VGLUT3 are expressed by CCK-containing basket cells, their decreased density in immunoreactivity in *stratum pyramidale* could represent a decreased CCK basket cell influence onto the cell bodies of pyramidal neurons. If so, this imbalance of inhibitory input within this domain could have a profound influence on the firing patterns of pyramidal neurons. This decreased inhibitory influence from CCK-expressing basket cell could be compensated for by the inhibitory tone of PV-expressing basket cells. However, it is not only the release of GABA, but also the amount released within a specific time frame that signifies the role of a specific interneuron with a network. Defining differences between PV and CCK-expressing basket cells are their firing rates [61], the ultrastructure of their synaptic inputs onto pyramidal cell somata [62] and their contributions to entraining the activity of specific subsets of pyramidal neurons [60]. It is this firing of subsets of pyramidal cells during different behavioural states that underlies the role of the hippocampus in different cognitive processes [63]. Thus, these anatomical data demonstrate the potential alterations in the molecular machinery that underpins pyramidal cell excitability, ushering in a potentially novel role of dystrophin in patterning the formation of specific subsets of synapses in addition to its well established role in anchoring neurotransmitter receptors [14].
